# Screening and Identification of Potential Prognostic Biomarkers in Adrenocortical Carcinoma

**DOI:** 10.3389/fgene.2019.00821

**Published:** 2019-09-11

**Authors:** Wen-Hao Xu, Junlong Wu, Jun Wang, Fang-Ning Wan, Hong-Kai Wang, Da-Long Cao, Yuan-Yuan Qu, Hai-Liang Zhang, Ding-Wei Ye

**Affiliations:** ^1^Department of Urology, Fudan University Shanghai Cancer Center, Shanghai, China; ^2^Department of Oncology, Shanghai Medical College, Fudan University, Shanghai, China

**Keywords:** adrenocortical carcinoma, bioinformatics analysis, biomarker, prognosis, network module

## Abstract

**Objective:** Adrenocortical carcinoma (ACC) is a rare but aggressive malignant cancer that has been attracting growing attention over recent decades. This study aims to integrate protein interaction networks with gene expression profiles to identify potential biomarkers with prognostic value *in silico*.

**Methods:** Three microarray data sets were downloaded from the Gene Expression Omnibus (GEO) database to identify differentially expressed genes (DEGs) according to the normalization annotation information. Enrichment analyses were utilized to describe biological functions. A protein–protein interaction network (PPI) of the DEGs was developed, and the modules were analyzed using STRING and Cytoscape. LASSO Cox regression was used to identify independent prognostic factors. The Kaplan–Meier method for the integrated expression score was applied to analyze survival outcomes. A receiver operating characteristic (ROC) curve was constructed with area under curve (AUC) analysis to determine the diagnostic ability of the candidate biomarkers.

**Results:** A total of 150 DEGs and 24 significant hub genes with functional enrichment were identified as candidate prognostic biomarkers. LASSO Cox regression suggested that *ZWINT*, *PRC1*, *CDKN3*, *CDK1* and *CCNA2* were independent prognostic factors in ACC. In multivariate Cox analysis, the integrated expression scores of the modules showed statistical significance in predicting **disease-free survival (DFS, *P* = 0.019) and overall survival (OS, *P* < 0.001)**. Meanwhile, ROC curves were generated to validate the ability of the Cox model to predict prognosis. The AUC index for the integrated genes scores was 0.861 (*P* < 0.0001).

**Conclusion:** In conclusion, the present study identifies DEGs and hub genes that may be involved in poor prognosis and early recurrence of ACC. The expression levels of *ZWINT*, *PRC1*, *CDKN3*, *CDK1* and *CCNA2* are of high prognostic value, and may help us understand better the underlying carcinogenesis or progression of ACC. Further studies are required to elucidate molecular pathogenesis and alteration in signaling pathways for these genes in ACC.

## Introduction

Adrenocortical carcinoma (ACC) is a rare endocrine malignancy with an annual incidence of 0.7–2.0 per million people, accounting for an estimated 0.02% of all cancers (Wajchenberg et al., 2000; Kebebew et al., 2006; Kerkhofs et al., 2013). Although comparatively uncommon, ACC patients often face aggressive progression, with merely less than 35% of patients surviving 5 years after initial diagnosis (Else et al., 2014). Currently, the preferred treatment regimen for ACC is surgical resection of the primary tumor (Fassnacht et al., 2013). However, almost half of ACC patients have disseminated metastasis, and approximately one-third of patients have locoregional metastases after surgery (Else et al., 2014). The first–line treatment, and the only ACC-specific medical therapy approved by the US Food and Drug Administration, is Mitotane, which is regularly used as an adjuvant agent in these patients (Else et al., 2014). Mitotane disrupts mitochondria and activates an apoptotic process (Poli et al., 2013). A major concern of the therapeutic management with Mitotane is the risk of toxicity, which may lead to severe adrenal insufficiency (Paragliola et al., 2018).

Accumulating evidence has demonstrated that gene expression levels and related pathways are involved in the carcinogenesis and progression of ACC. For example, the most frequent alterations observed in ACC are overexpression of insulin-like growth factor 2 (*IGF-2*) (Gicquel et al., 2001; Giordano et al., 2003; de Fraipont et al., 2005) and constitutive activation of the Wnt/β-Catenin pathway (Gaujoux et al., 2011). Despite these encouraging advances in ACC clinical strategies, only a minority of patients receive any significant survival benefit because of a lack of effective therapeutic strategies (Mohan et al., 2018). Therefore, it is crucial to understand the underlying molecular mechanisms involved in the carcinogenesis, proliferation and recurrence of ACC and thus develop effective diagnostic and therapeutic strategies.

Over the last decade, microarray technologies and bioinformatic analysis have been widely used to detect comprehensive mRNA expression levels, which have assisted in identifying the differentially expressed genes (DEGs) and functional pathways involved in the tumorigenesis and progression of ACC. However, because of the rarity of this tumor, there has been a problem in identifying potential markers to differentiate ACC from other renal neoplasms, and thus guiding potential treatment strategy. In the present study, three mRNA microarray datasets were downloaded from GEO database and analyzed to obtain DEGs between cancer tissues and adjacent normal tissues. Subsequently, functional pathway enrichment analyses were implemented to further understand the molecular mechanisms underlying carcinogenesis. The protein–protein interaction (PPI) network reveals the functions of all proteins and the importance of these interactions with regards to biological processes, molecular functions, and signal transduction (Sharan et al., 2007; Wu et al., 2009; Bapat et al., 2010). This may provide insights into the mechanisms of generation or development of diseases.

To investigate candidate biomarkers in tumor tissue and to define their value in ACC patients, this work focuses on analyzing the gene expression profiles, revealing the underlying biological interaction networks and assessing their prognostic value. We hypothesize that the oncogenic activity of significant hub genes correlates with poor prognosis, and might reveal potential prognostic markers and therapeutic targets for ACC.

## Materials and Methods

### Raw Biological Microarray Data

The raw DNA microarray data were obtained from GEO (http://www.ncbi.nlm.nih.gov/geo) (Edgar et al., 2002) for patients with ACC. Corresponding genes converted into the probes were converted into symbols according to the annotation information in the platform. Three chip data sets GSE14922, GSE19750 and GSE90713 (4 normal and 4 ACC samples in GSE14922, 4 normal and 44 ACC samples in GSE19750, and 5 normal and 58 ACC samples in GSE90713) were downloaded from GEO (Agilent GPL6480 platform, Affymetrix GPL570 platform and Affymetrix GPL15270 platform, respectively).

### Normalization and Elucidation of DEGs

DNA microarray analysis begins with preprocessing and normalization of raw biological data. This process removes noise from the biological data and ensures its integrity. Next, background correction of probe data, normalization, and summarization were executed by robust multi-array average analysis algorithm17 in affy package of R.

The DEGs between ACC and non-cancerous samples were screened and identified across experimental conditions. Delineating parameters such as adjusted P-values (adj. P), Benjamini and Hochberg false discovery rate (FDR) and fold change were utilized for filtering of DEGs and applied to provide a balance between discovery of statistically significant genes and limitations of false-positives. Probe sets without corresponding gene symbols or genes with more than one probe set were removed or averaged. Log_2_FC (fold change) > 1 and adj. P-value <0.01 were considered statistically significant.

### Functional Enrichment of DEGs

Discerning the role of DEGs in ACC, biological attributes including biological processes (BP), molecular functions (MF), and cellular components (CC) were extracted from Gene Ontology (GO) enrichment analysis (Ashburner et al., 2000). Kyoto Encyclopedia of Genes and Genomes (KEGG) (Kanehisa et al., 2016) is a database resource for understanding high-level functions and biological systems from large-scale molecular datasets generated by high-throughput experimental technologies. The online Database for Annotation, Visualization and Integrated Discovery (DAVID; https://david-d.ncifcrf.gov/summary.jsp Version 6.8) was used to explore the role of development-related signaling pathways in ACC (Huang et al., 2007). P-value < 0.05 was considered statistically significant. GO enrichment was analyzed and displayed using a bubble chart.

### PPI Network Construction and Module Analysis

In the present study, the Search Tool for the Retrieval of Interacting Genes (STRING; http://string-db.org) (version 10.0) online database was used to predict PPI network of DEGs and analyze the functional interactions between proteins (Franceschini et al., 2013). An interaction with a combined score >0.4 was considered statistically significant.

Cytoscape (version 3.5), an open source bioinformatics software platform, was used to visualize molecular interaction networks (Smoot et al., 2011). Molecular Complex Detection (MCODE) (version 1.4.2) is a plug-in for Cytoscape used for clustering a given network based on topology to find densely connected regions (Bandettini et al., 2012). MCODE could identify the most significant module in the PPI networks with selection as follows: MCODE scores >5, degree cut-off = 2, node score cut-off = 0.2, Max depth = 100 and k-score = 2. Subsequently, the KEGG and GO analyses for genes in this module were performed using DAVID.

### Hub Genes Selection and Analysis

The hub nodes of network with connectivity degrees >10 were identified. A network of the 24 genes and their co-expression genes was analyzed using cBioPortal (http://www.cbioportal.org) online platform (Cerami et al., 2012). ClueGO is a Cytoscape plug-in that visualizes the non-redundant biological terms for large clusters of genes in a functionally grouped network (Bindea et al., 2009). The biological process from GO and KEGG pathway analysis of hub genes was performed and visualized using ClueGO (version 2.5.3) and CluePedia (version 1.5.3), a functional extension of ClueGO, plug-in of Cytoscape (Bindea et al., 2013). Potential coexpression relationship between the 24 hub genes and possible prognostic value are shown in a heat map.

### Statistical Analysis

Phenotype and expression profiles of hub genes in 76 ACC patients from TCGA were analyzed and displayed. Clinical and pathological parameters of the cohort were summarized. Expression of hub genes was respectively identified as binary variables (high vs. low) referring to median expression of each hub gene in the TCGA cohort. Then, a LASSO Cox regression model was constructed to find independent prognostic factors. The significant hub gene expression profiles of common neoplasm were analyzed and displayed using Oncomine online database (http://www.oncomine.com) (Giordano et al., 2003; Giordano et al., 2009).

The Kaplan–Meier method was applied to analyze survival differences between groups. The primary end point was overall survival (OS) for patients, which was evaluated from the date of first therapy to the date of death or last follow-up. Disease-free survival (DFS), as the secondary end point, was the length of time from the initiation of curative treatment to the date of progression or the start date of a second-line treatment or the date of death, whichever occurred first. The follow-up duration was estimated using the Kaplan-Meier method with 95% confidence intervals (95%CI) and log-rank test in separate curves. Univariate analyses were performed with Cox logistic regression models to find independent variables, including age at diagnosis, gender, laterality, TNM stage, pathologic stage, mitotic rate, invasion of tumor capsule, sinusoid invasion, necrosis, Weiss score, new tumor event after first treatment and integrated expression score. Parameters with P-value less than 0.1 were enrolled in multivariate Cox regression analyses of DFS and OS in “Back-LR” method. Integrated score was identified as the sum of the weight of each significant hub gene. X-tile software was utilized to take the cut-off value. All hypothetical tests were two-sided and P-values less than 0.05 were considered significant in all tests. The receiver operating characteristic curve (ROC) was constructed by predicting the probability of a diagnosis being of high or low integrated score of significant hub gene expression. Area under curve (AUC) analysis was performed to determine the diagnostic ability.

### Sensitivity Analysis of Chip Datasets

In this study, GSE14922 only contains 4 ACC patients and 4 normal patients, while datasets 2 and 3 have 44 and 58 ACC samples respectively. To avoid penitential bias and see what other significant genes may have been missed, the analysis was re-run without GSE14922. Prognostic values of DEGs were then also tested against the TCGA validation cohort.

### Data Processing of Gene Set Enrichment Analysis (GSEA)

TCGA database was implemented with the GSEA method using the Category version 2.10.1 package. For each separate analysis, Student’s-t-test statistical score was performed in consistent pathways, and the mean of the differential expression genes was calculated. A permutation test of 1000 times was used to identify the significantly changed pathways. The adjusted P values (adj. P) using Benjamini and Hochberg (BH) false discovery rate (FDR) method by default were applied to correct the occurrence of false positive results (Subramanian et al., 2005). The significant related genes were defined with an adj. P less than 0.01 and FDR less than 0.25. Statistical analysis and graphical plotting were conducted using R software (Version 3.3.2).

## Results

This study consisted of three stages. In the first stage, we assessed DEGs using three datasets hosted on the GEO platform. In the second stage, coexpression, functional annotation of hub genes and patient survival analysis were carried out. In the third stage, the most significant hub genes were selected, evaluated and integrated to predict their prognostic value.

### Identification of DEGs in ACC

**Figure 1:**
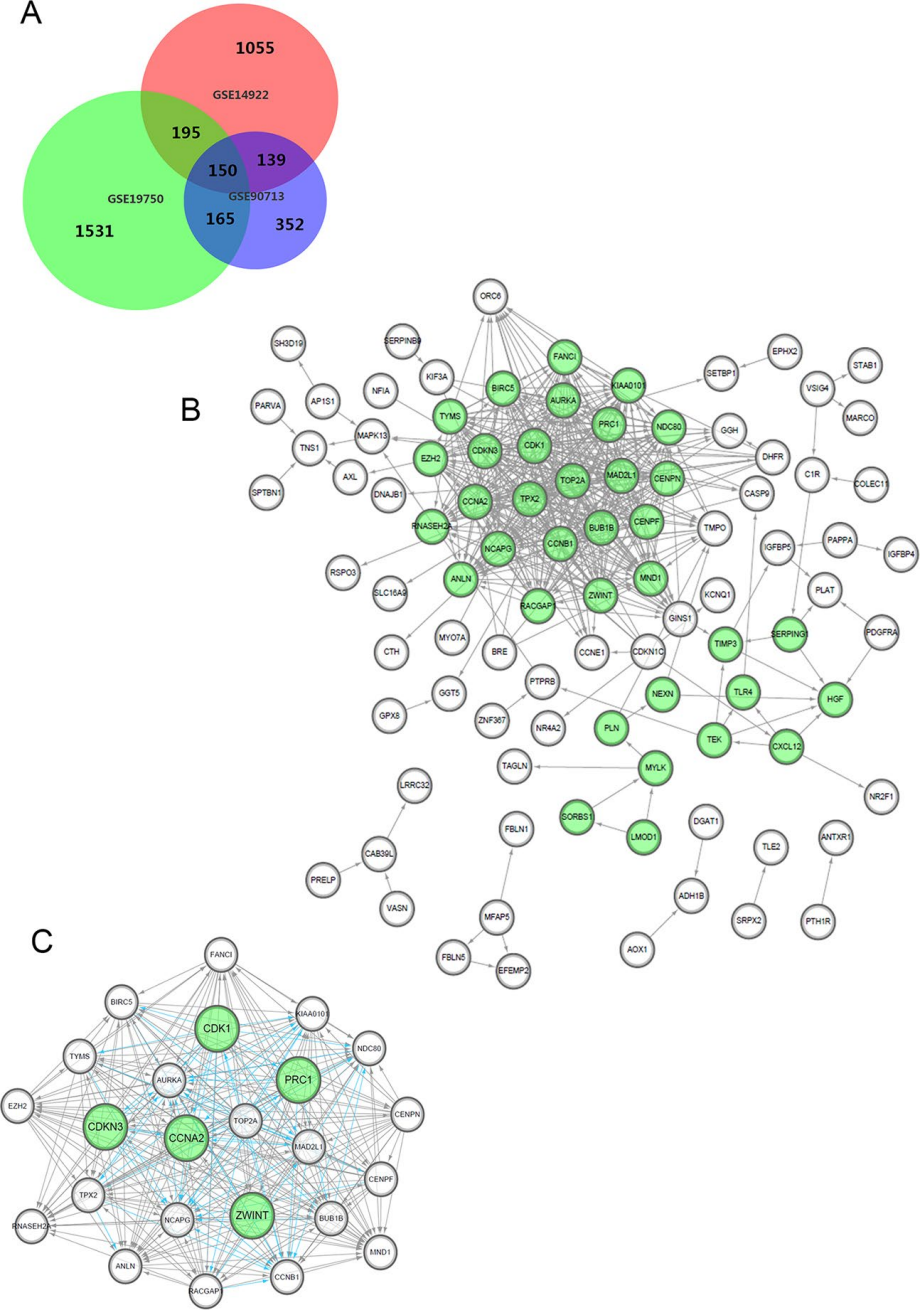
Venn diagram, PPI network and the most significant module of DEGs. **(A)** DEGs were selected with a fold change >2 and P-value <0.01 among the mRNA expression profiling chip datasets GSE14922, GSE19750 and GSE90713. The 3 datasets show an overlap of 150 genes in the Venn diagram. **(B)** The PPI network of DEGs was constructed using Cytoscape. **(C)** The most significant module was obtained from PPI network with 24 nodes. Significant edges are marked in light blue with a K-score >0.800.

After standardization and identification of the microarray results, the DEGs (1,804 probe samples with 1,539 DEGs in GSE14922, 2,454 probe samples with 2,040 DEGs in GSE19750 and 1,216 probe samples with 806 DEGs in GSE90713) were determined to be significant based on the analysis and the statistical parameters of the data processing steps. The overlap among the three datasets included 150 significant genes and is displayed in the Venn diagram in **Figure 1A**.

### GO and KEGG Enrichment Assessment of DEGs

To analyze the biological classification of the DEGs, functional and pathway enrichment analyses were performed using DAVID. As shown in **Supplementary Figure 1**, gene ontology (GO) analysis indicated that changes in the biological processes of the DEGs were significantly associated with the mitotic cell cycle, cell cycle process, movement of cells or subcellular components and cell locomotion activity. Changes in molecular function were mostly enriched in growth factor binding, kinase activity, extracellular matrix structure constituents and insulin-like growth factor binding. Changes in cellular components were mainly enriched in the chromosome, centromeric region, extracellular region, actin cytoskeleton and mitotic spindle. KEGG pathway analysis revealed that the DEGs were mainly enriched in cell cycle, progesterone-mediated oocyte maturation, oocyte meiosis, arachidonic acid metabolism and the p53 signaling pathway, summarized in **Table 1**.

**Table 1:** KEGG pathways enrichment analysis of DEGs in ACC samples.

Term	Description	Count in gene set	P value
Has04110	Cell cycle	7	7.01E-04
Has04914	Progesterone-mediated oocyte maturation	5	6.05E-03
Has04114	Oocyte meiosis	5	0.01757
Has00590	Arachidonic acid metabolism	4	0.01758
Has04115	p53 signaling pathway	4	0.02312
Has05133	Pertussis	4	0.02667
Has06161	Hepatitis B	5	0.04010
Has00380	Tryptophan metabolism	3	0.04228

KEGG, Kyoto Encyclopedia of Genes and Genomes; DEGs, differentially expressed genes; ACC, adrenocortical carcinoma.

### PPI Network Establishment and Module Analysis

We constructed the PPI network of the DEGs (**Figure 1B**) and subsequently found the most significant module penal using a Cytoscape plugin (**Figure 1C**). The enrichment profiles from DAVID functional analyses of the 24 hub genes suggested that the hub genes in this module were primarily enriched in cell cycle phase, M phase, the mitotic cell cycle and mitosis (**Table 2**).

**Table 2:** GO and KEGG pathways enrichment analysis of DEGs in the most significant module.

Term	Description	Count in gene set	P value
GO:0022403	Cell cycle phase	16	6.836E-19
GO:0022402	Cell cycle process	17	1.154E-18
GO:0000279	M phase	15	1.898E-18
GO:0000278	Mitotic cell cycle	15	9.924E-18
GO:0007067	Mitosis	13	6.478E-17
GO:0000280	Nuclear division	13	6.477E-17
GO:0000087	M phase of mitotic cell cycle	13	8.063E-17
GO:0005819	Spindle	9	4.531E-11
GO:0015630	Microtubule cytoskeleton	11	4.532E-9
GO:0005694	Chromosome	10	3.922E-8
hsa04110	Cell cycle	5	2.268E-5
hsa04914	Progesterone-mediated oocyte maturation	4	2.461E-4
hsa04114	Oocyte meiosis	4	5.097E-4

GO, Gene Ontology; KEGG, Kyoto Encyclopedia of Genes and Genomes; DEGs, differentially expressed genes.

### Hub Gene Selection and Analysis

**Figure 2:**
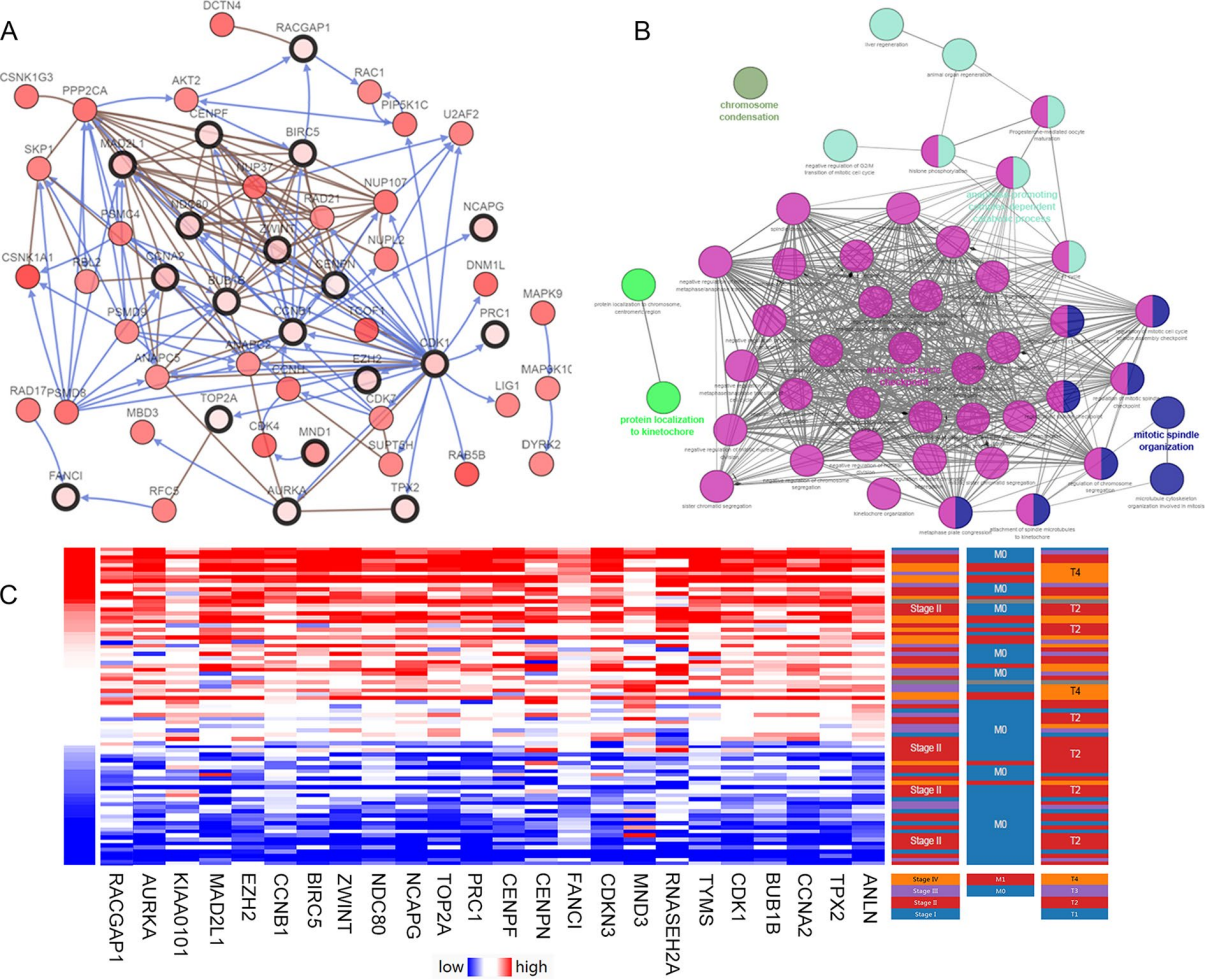
Interaction network and biological process analysis of the hub genes. **(A)** Hub genes and their co-expression network were analyzed using cBioPortal. Nodes with bold black outline represent hub genes. Nodes with thin black outline represent the co-expression genes. **(B)** The biological process analysis of hub genes was constructed using ClueGO. Different color of nodes refers to the functional annotation of ontologies. Corrected P-value < 0.01 was considered statistically significant. **(C)** Hierarchical partitioning of 24 hub genes was obtained from DNA microarrays. It represent the level of expression of 24 genes across a number of comparable samples with high expression samples marked in red and low in blue.

After statistical selection, the significant hub nodes of the network included RACGAP1, AURKA, KIAA0101, MAD2L1, ZEH2, CCNB1, BIRC5, ZWINT, NDC80, NCAPG, TOP2A, PRC1, CENPF, CENPN, FANCI, CDKN3, MND1, RNASEH2A, TYMS, CDK1, BUB1B, CCNA2, TPX2 and ANLN. A visual network of the 24 genes and their coexpressed genes was set up (**Figure 2A**). The GO biological processes and KEGG functional annotation analysis of the hub genes are shown in **Figure 2B**. The detailed functional notes and classification pie charts are provided in the **Supplementary Figure 2**. Of the GO biological processes, 66.67% of terms belonged to the mitotic cell cycle checkpoint, 15.79% to mitotic spindle organization, 12.28% to anaphase-promoting complex-dependent catabolic processes, 3.51% to protein localization to kinetochore, and 1.75% to chromosome condensation. A heat map shows that a potential coexpression relationship may exist between the 24 hub genes, which could suggest they have value for prognostic prediction (**Figure 2C**).

### Clinicopathological Statistical Analysis

**Figure 3:**
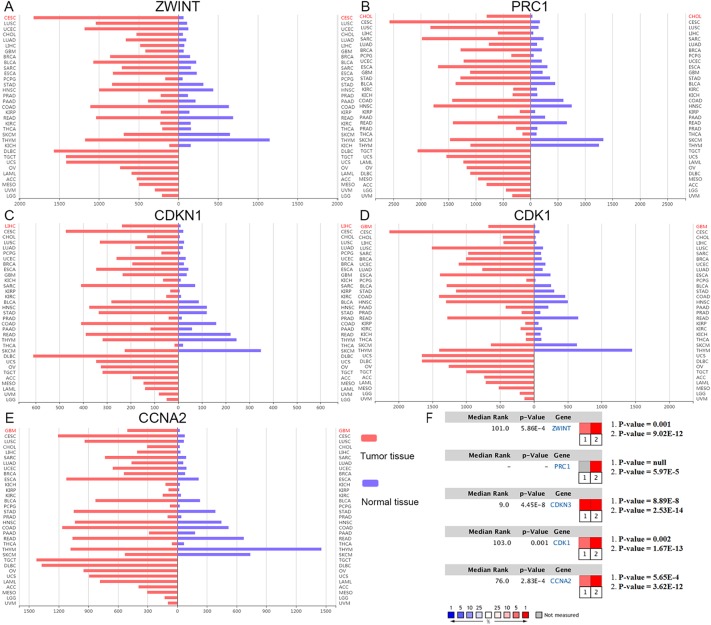
Oncomine expression analysis of cancer vs. normal tissue. **(A**–**E)** Histogram of *ZWINT*, *PRC1*, *CDKN1*, *CDK1* and *CCNA2* gene expression in total tumor spectrum samples vs. normal tissues. **(F)** Documented expression of significant molecular score in ACC samples. 1. Giordano, T.J., et al., Distinct transcriptional profiles of adrenocortical tumors uncovered by DNA microarray analysis. Am J Pathol, 2003 (Giordano et al., 2003). 2. Giordano, T.J., et al., Molecular classification and prognostication of adrenocortical tumors by transcriptome profiling. Clin Cancer Res, 2009 (Giordano et al., 2009).

**Figure 4:**
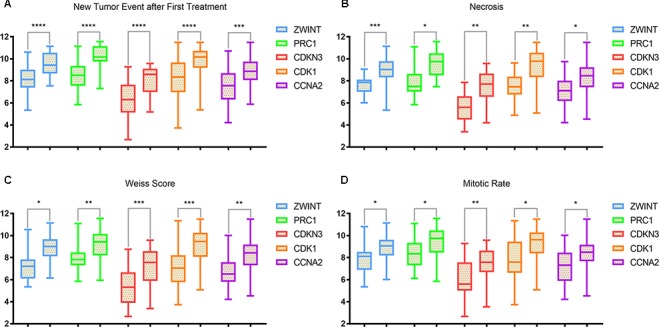
Elevated expression patterns (*ZWINT* presents in light blue, PRC1 in light green, *CDKN3* in maroon, *CDK1* in orange and *CCNA2* in purple) were significantly associated with **(A)** new tumor events after first treatments, **(B)** necrosis, **(C)** Weiss score and **(D)** mitotic rate >50 high power field (HPF).

The clinical and pathological parameters from phenotype and expression profiles of the hub genes in 76 ACC patients from The Cancer Genome Atlas (TCGA) are summarized in **Table 3**. Each hub gene was classified into dichotomous variables according to the median expression in the analysis. Subsequently, the univariate survival analysis of the hub genes was performed using a Kaplan–Meier curve. Apart from *MND1*, ACC patients with elevated expression of the other 23 hub genes showed significantly worse OS and DFS (**Supplementary Figure 3**). LASSO Cox regression suggested that *ZWINT*, *PRC1*, *CDKN3*, *CDK1* and *CCNA2* are significant weighted prognostic factors, and that an integrated gene panel may serve as an independent penal in ACC samples. The five significant hub gene expression profiles showed significantly elevated expression in tumor tissues compared with the corresponding normal tissues (**Figures 3A–E**). In addition, differential analysis from the ONCOMINE online database of tumor and normal tissue in two cohorts indicated that *ZWINT*, *PRC1*, *CDKN3*, *CDK1* and *CCNA2* were highly expressed in ACC samples (**Figure 3F**). Elevated expression patterns were significantly associated with distant metastasis, necrosis, Weiss score and mitotic rate of >5 mitoses per 50 high power fields (HPF), plotted in **Figure 4**.

**Table 3:** Clinicopathologic characteristics of 76 ACC patients from TCGA database.

Characteristics	Entire cohort (N = 76)
N (%)	
Age, years	
≤57	54 (71.1)
>58	22 (28.9)
Gender	
Male	30 (39.5)
Female	46 (60.5)
Germline testing performed	
Present	12 (18.2)
Absent	54 (81.1)
Laterality	
Left	42 (55.3)
Right	34 (44.7)
pT stage	
T1 – T2	50 (65.8)
T3 – T4	26 (34.2)
pN stage	
N0	68 (89.5)
N1	8 (10.5)
M stage	
M0	62 (81.6)
M1	14 (18.4)
Pathologic stage	
I – II	46 (60.5)
III – IV	30 (39.5)
Histological type	
Myxiod	1 (1.3)
Oncocytic	3 (3.9)
Usual	72 (94.7)
Mitotic rate	
≤5/50 HPF	26 (38.8)
>5/50 HPF	41 (61.2)
Invasion of tumor capsule	
Absent	30 (42.9)
Present	40 (57.1)
Sinusoid invasion	
Absent	34 (58.6)
Present	24 (41.4)
Necrosis	
Absent	17 (23.9)
Present	54 (76.1)
Weiss score	
≤3	17 (22.0)
>4	47 (78.0)
Persistent distant metastasis	
Absent	56 (73.7)
Present	20 (26.3)

ACC, adrenocortical carcinoma; TCGA, the Cancer Genome Atlas; HPF, high power field.

### Cox Regression Analyses and Survival Outcomes of the Cohorts

In this study, the integrated expression score was identified as the sum of the weight of each binary gene expression. In univariate models, traditional prognostic factors, specifically T stage, M stage and pathologic stage, were significantly correlated with DFS (*P* < 0.001) and OS (*P* < 0.001) in ACC patients. Importantly, in univariate Cox regression analyses of DFS, subgroups of integrated expression score (High vs. Low) showed that integrated gene expression amplification significantly correlated with poor DFS (P < 0.001) for ACC patients. In addition, mitotic rate (≤5/50 HPF vs. >5/50 HPF) (*P* = 0.022), necrosis (**Present** vs. **Absent**) (*P* = 0.011), Weiss score(≤3 vs. >3) (*P* = 0.008) and new tumor event (**Present** vs. **Absent**) (*P* < 0.001) were correlated with poor DFS. In univariate Cox regression analyses of OS, invasion of tumor capsule (**Present** vs. **Absent**) (*P* = 0.013), sinusoid invasion (**Present** vs. **Absent**) (*P* = 0.011), necrosis (**Present** vs. **Absent**) (*P* = 0.026) and new tumor event (**Present** vs. **Absent**) (*P* < 0.001) were associated with shorter OS. However, in multivariate prognostic analysis, new tumor event after first treatment (*P* < 0.001) and integrated expression score (*P* = 0.019) were statistically significant parameters in predicting DFS (**Table 4**). Age at diagnosis (*P* = 0.003), M stage (*P* = 0.033), new tumor event after first treatment (*P* = 0.013) and integrated expression score (*P* < 0.001) were significantly associated with shorter OS (**Table 5**).

**Table 4:** Univariate and multivariate Cox regression analyses of DFS in 76 enrolled ACC patients.

	Univariate analysis		Multivariate analysis	
Covariates	HR (95%CI)	P value	HR (95%CI)	P value
Age at diagnosis (≤57 years vs. >58 years)	1.703 (0.819 – 3.541)	0.154		
Gender (male vs. female)	0.977 (0.477 – 2.002)	0.950		
Laterality (left vs. right)	0.771 (0.381 – 1.563)	0.471		
T stage (T1-T2 vs. T3-T4)	3.846 (1.841 – 8.034)	**<0.001**		
N stage (N0 vs. N1)	2.151 (0.820 – 5.641)	0.119		
M stage (M0 vs. M1)	3.104 (1.471 – 6.546)	**0.003**	2.193 (0.977 – 4.921)	0.057
Pathologic stage (I - II vs. III - IV)	3.937 (1.853 – 8.364)	**<0.001**		
Mitotic rate (≤5/50 HPF vs. >5/50 HPF)	2.851 (1.164 – 6.984)	**0.022**		
Invasion of tumor capsule (Present vs. Absent)	2.074 (0.965 – 4.455)	0.062		
Sinusoid invasion (Present vs. Absent)	1.516 (0.678 – 3.389)	0.311		
Necrosis (Present vs. Absent)	6.501 (1.542 – 27.404)	**0.011**		
Weiss score (≤3 vs. >3)	2.816 (1.303 – 6.085)	**0.008**		
New tumor event (Present vs. Absent)	16.642 (5.673 – 48.822)	**<0.001**	9.041 (2.983 – 27.234)	**<0.001**
Integrated expression score (High vs. Low)	7.819 (3.569 – 17.114)	**<0.001**	2.767 (1.185 – 6.460)	**0.019**

DFS, disease-free survival; ACC, clear cell renal cell carcinoma; HR, hazard ratio; CI, confidence interval; HPF, high power field.

*Multivariate Cox regression analyses of DFS in 76 enrolled ACC patients was run in “Back-LR” method. Statistically significant is considered as P value less than 0.05, indicated in bold.

**Table 5:** Univariate and multivariate Cox regression analyses of OS in 76 enrolled ACC patients.

	Univariate analysis		Multivariate analysis*	
Covariates	HR (95%CI)	P value	HR (95%CI)	P value
Age at diagnosis (≤57 years vs. >58 years)	1.957 (0.913 – 4.196)	0.085	4.959 (1.744 – 14.098)	**0.003**
Gender (male vs. female)	0.996 (0.466 – 2.127)	0.991		
Laterality (left vs. right)	1.262 (0.591 – 2.695)	0.548		
T stage (T1-T2 vs. T3-T4)	10.693 (4.276 – 28.110)	**<0.001**		
N stage (N0 vs. N1)	0.451 (0.171 – 1.191)	0.108		
M stage (M0 vs. M1)	7.340 (3.300 – 16.327)	**<0.001**	3.045 (1.094 – 8.477)	**0.033**
Pathologic stage (I - II vs. III - IV)	7.157 (3.023 – 16.941)	**<0.001**		
Mitotic rate (≤5/50 HPF vs. >5/50 HPF)	1.708 (0.743 – 3.926)	0.208		
Invasion of tumor capsule (Present vs. Absent)	3.015 (1.257 – 7.231)	**0.013**		
Sinusoid invasion (Present vs. Absent)	3.069 (1.297 – 7.262)	**0.011**		
Necrosis (Present vs. Absent)	5.135 (1.214 – 21.725)	**0.026**		
Weiss score (≤3 vs. >3)	3.467 (1.136 – 10.577)	0.307		
New tumor event (Present vs. Absent)	5.833 (2.351 – 14.473)	**<0.001**	3.609 (1.305 – 9.980)	**0.013**
Integrated expression score (High vs. Low)	18.892 (6.830 – 52.255)	**<0.001**	18.719 (5.122 – 68.415)	**<0.001**

OS, overall survival; ACC, adrenocortical carcinoma; HR, hazard ratio; CI, confidence interval; HPF, high power field.

*Multivariate cox regression analyses of DFS in 76 enrolled ACC patients was run in “Back-LR” method. Statistically significant is considered as P value less than 0.05, indicated in bold.

**Figure 5:**
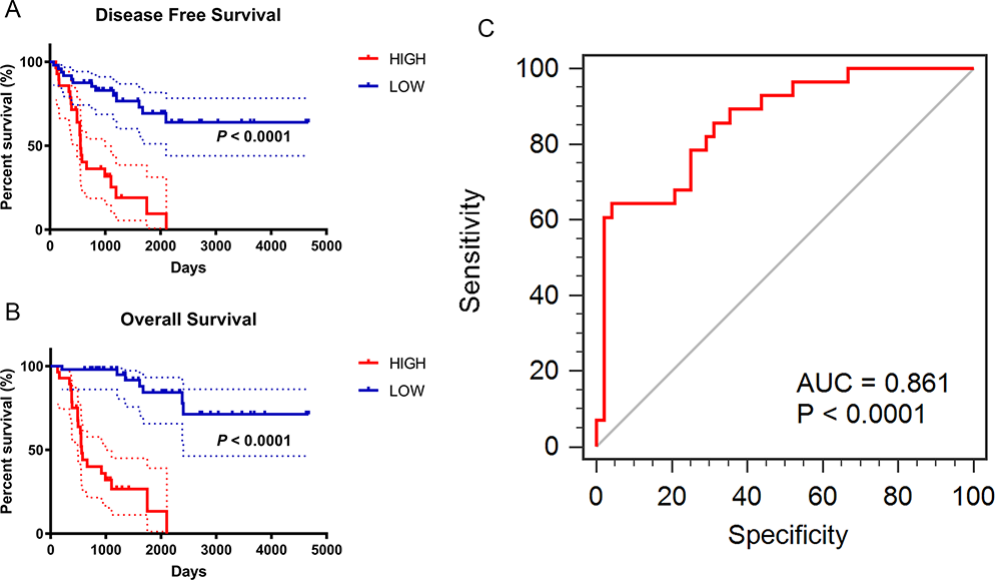
Prognostic and diagnostic value of integrated significant molecular score in ACC samples after LASSO Cox regression. **(A**–**B)** Kaplan–Meier method was used to perform the significant survival outcomes (DFS: *P* < 0.0001; OS: *P* < 0.0001). **(C)** ROC curves of the integrated models were synchronously plotted to predict diagnosis probability. Red line represents integrated expression score with AUC of 0.861 (*P* < 0.0001).

*Multivariate Cox regression analyses of OS in 76 enrolled ACC patients was run in “Back-LR” method. After integrating all the significant gene expression profiles in the Cox regression models, the Kaplan–Meier method was used to determine the significant survival outcomes (DFS: *P* < 0.0001; OS: *P* < 0.0001), shown in **Figures 5A, B**. Meanwhile, ROC curves were generated to validate the ability of the logistic model to predict prognosis. The AUC index for the integrated gene scores was 0.861 (*P* < 0.0001) (**Figure 5C**).

### Sensitivity Analysis of Chip Datasets

To avoid penitential bias and see what other significant genes may have been missed, two datasets GSE19750 and GSE90713 were enrolled to re-run the analysis. The overlap among the two datasets, which includes 315 differential expressed genes (DEGs), is displayed in the Venn diagram (**Supplementary Figure 4A**). A PPI network of the new DEGs was constructed in **Supplementary Figure 4B**. Subsequently, we selected the most significant module penal using M-CODE, a plug-in of Cytoscape, and found 31 hub genes including *NDC80*, *MND1*, *MAD2L1*, *UBE2C*, *NCAPG*, *GINS1*, *CENPN*, *CDKN3*, *CCNA2*, *ZWINT*, *BIRC5*, *KIAA0101*, *TOP2A*, *BUB1B*, *CCNB1*, *AURKA*, *SMC2*, *ATAD2*, *PRC1*, *TPX2*, *CDK1*, *RACGAP1*, *TYMS*, *ANLN*, *PRIM1*, *NUSAP1*, *CENPF*, *SPAG5*, *SMC4*, *EZH2*, *FANCI*. Interestingly, five significant DEGs we have focused on (*ZWINT*, *PRC1*, *CDKN3*, *CDK1*, *CCNA2*) still consist of this new module penal (**Supplementary Figure 4C**), indicating a good stability of our molecular model. Eight different DEGs are found different from these in three-chipset study, including *UBE2C*, *GINS1*, *SMC2*, *ATAD2*, *PRIM1*, *NUSAP1*, *SPAG5*, *SMC4*. Kaplan-Meier method was used to analyze mRNA expression level of 8 hub genes in TCGA cohort, which also showed statistically significant correlation with progressive progression and poor prognosis (**Supplementary Figure 5**).

### Significant Genes and Pathways Obtained by GSEA

**Figure 6:**
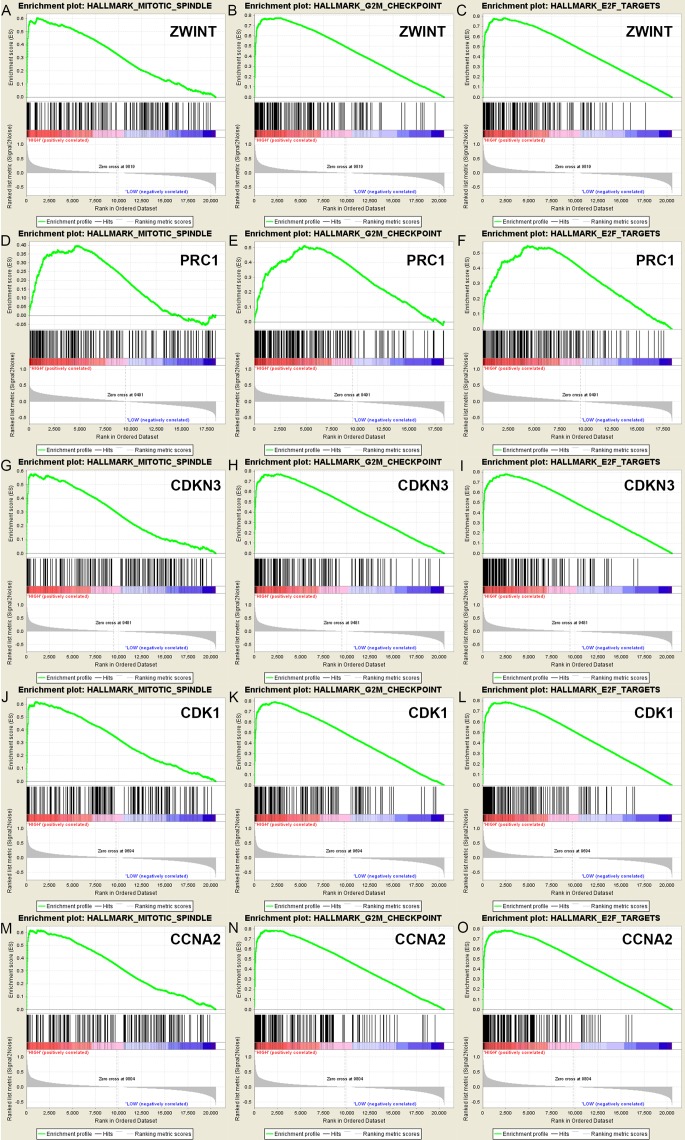
A total of 100 significant genes were obtained from GSEA with positive and negative correlation. GSEA was used to perform hallmark analysis in *ZWINT*, *PRC1*, *CDKN3*, *CDK1* and *CCNA2*, respectively. Results of GESA suggested that **(A**–**C)** *ZWINT*, **(D**–**F)** *PRC1*, **(G**–**I)** *CDKN3*, **(J**–**L)** *CDK1*, **(M**–**O)** *CCNA2* significantly involved in the same hallmarks pathways including mitotic spindle, G2M checkpoint and E2F targets.

A total of 100 significant genes were obtained by gene set enrichment analysis (GSEA) with positive and negative correlation. Importantly, GSEA was used to perform hallmark analysis for *ZWINT*, *PRC1*, *CDKN3*, *CDK1* and *CCNA2*. This suggested that the most involved significant pathways included mitotic spindle, G2M checkpoint and E2F targets. The details are shown in **Figure 6**.

## Discussion

Adrenocortical carcinoma (ACC) is a rare but aggressive cancer, with a typically high incidence in children with a *TP53* germline mutation (Fassnacht et al., 2013). The Wnt/β-catenin pathway and IGF-2 signaling have been confirmed as altered signaling pathways in ACC patients, while increasing data indicate that the available evidence is inadequate for malignant phenotype and poor prognosis (Berthon et al., 2010; Heaton et al., 2012), especially for the diagnosis of low-grade ACC confined to the adrenal gland (Mete et al., 2018). Although there are diagnostic and prognostic molecular tests for ACC such as the IGF-2, Ki-67, p53, BUB1B, PBK, HURP, NEK2, DAX, Wnt/β-catenin and PI3K signaling pathways, they remain largely unutilized in morphologic assessment coupled with ancillary diagnostic and prognostic modeling of ACC (Mete et al., 2018). Therefore, the major molecular mechanisms in the pathogenesis and progression are poorly understood. In 2003 and 2009, Giordano et al. performed unsupervised cluster analyses of transcriptome data to identify subgroups with different prognoses (Giordano et al., 2003; Giordano et al., 2009). These two studies laid the foundation for the molecular classification and prognostication of adrenocortical tumors and also provided a rich source of potential diagnostic and prognostic markers. Still, most cases of ACC were initially diagnosed with highly aggressive progression but were not candidates for curative therapies. Hence, potential biomarkers for diagnosis and treatment with high efficiency are urgently demanded.

Currently, microarray technology enables comprehensive mRNA expression profiling in ACC and can identify and investigate new biomarkers involved in tumorigenesis. A total of 150 DEGs and 24 hub genes were identified by microarray data analysis. GO and KEGG enrichment analysis showed association to the cell cycle, especially mitotic cycle checkpoint, mitotic spindle and oocyte meiosis, which was the most significant annotated function. Furthermore, among the 24 hub genes, the most significant molecular prognostic model integrated *ZWINT*, *PRC1*, *CDKN3*, *CDK1* and *CCNA2*. Importantly, after reintegrating the weight of each gene, the new score was statistically the most significant parameter in both univariate and multivariate regression analysis. The gene set enrichment analysis (GSEA) method was used to visualize the significant signaling pathway analysis of *ZWINT*, *PRC1*, *CDKN3*, *CDK1* and *CCNA2*.

ZW10 interactor (*ZWINT*), an interactor with ZW10, plays a vital role in rectifying incorrect centromere-microtubule attachment and regulating the miototic spindle checkpoint (Starr et al., 2000). Increased expression of *ZWINT* correlates with poor outcomes in human malignancies, including prostate, ovarian, bladder and lung cancers (Bhattacharjee et al., 2001; Endoh et al., 2004; Urbanucci et al., 2012; Xu et al., 2016). These new findings encourage further investigation of the potential clinical significance in human malignancies, yet the prognostic value of *ZWINT* in ACC has rarely been reported.

Protein Regulator of cytokinesis 1 (*PRC1*) protein is located in the nucleus. It is highly expressed in S and G2/M phases and shows an obvious drop in the G1 phase of the cell cycle (Freedland et al., 2013). During anaphase, it dynamically locates with the mitotic spindle and localizes to the cell midbody (Wu et al., 2018). Increasing evidence suggests that PRC1 may be involved in a cancer-specific manner, because of its negative correlation with p53 and overexpression in p53-defective cells *in vitro* (Li et al., 2004). In addition, Chen et al. and Zhan et al. demonstrated that *PRC1* contributes to tumorigenesis by regulating the Wnt/β-catenin signaling pathway in a positive feedback loop (Chen et al., 2016; Zhan et al., 2017), in which carcinogenesis and progression may feasibly be mediated in ACC.

Cyclin-dependent kinase inhibitor 3 (*CDKN3*) is part of the dual-specificity protein phosphatase family that dephosphorylates CDK2/CDK1 kinase and other cytokines (Hannon et al., 1994). Interestingly, a relationship between elevated *CDKN3* expression and poor prognosis has been reported in many cancers by modulation of the cell cycle, mitotic spindle or p53 pathways (Berumen et al., 2014; Fan et al., 2015). A previous study has distinguished five genes modeling ACC using *TOP2A*, *NDC80*, *CEP55*, *CDKN3* and *CDK1*, which may be utilized to form a board of progressive and predictive biomarkers for ACC for clinical purpose (Xiao et al., 2018). Thus, it is inferred that *CDKN3* may be an oncogene in human ACC.

Cyclin dependent kinase 1 (*CDK1*) is a catalytic subunit of a highly conserved protein and is involved in many biological processes including cell cycle control, DNA damage repair, and checkpoint transcription (Skotheim et al., 2008; Enserink and Kolodner, 2010). *CDK1* plays an important regulatory role in the control of the eukaryotic cell cycle by modulating the centrosome cycle (Asghar et al., 2015). It has been previously reported that inhibition of *CDK1* could serve as a therapeutic target *via* microRNA-7 for ACC samples *in vivo* (Glover et al., 2015). Meanwhile, CDC2, sharing approximately 63% amino-acid homology with CDK1, was found to be dysregulated in the cell cycle or retinoic acid signaling pathway by meta-analysis of genomic profiling data of adrenocortical tumors (Szabo et al., 2010).

Cyclin-A2 (*CCNA2*) belongs to a highly conserved cyclin family whose members function as regulators of the cell cycle. This protein interacts with CDK2 during G1/S and in G2/M phase, therefore promoting cell cycle transition (Pagano et al., 1992). There is accumulating evidence suggesting a role for CCNA2 in tumorigenesis of human malignancies. Kim et al. identified an SNP (rs769236) at the *CCNA2* promoter that may be significantly associated with an increased risk of colon, liver and lung cancers (Kim et al., 2011). In addition, a significant delay in liver tumor formation was observed in mice with CCNA2-deficient hepatocytes (Gopinathan et al., 2014). As well as a prognostic value for *CDK1* in ACC (Xiao et al., 2018), the mitotic checkpoint regulator *CCNA2* may combine with other cell-cycle coding genes and be involved in aberrant regulation of the cell cycle network. It has not been evaluated whether this could be an effective approach to ACC treatment.

Our study represents the first attempt to construct a gene regulatory network incorporating DEGs and functional annotation of hub genes in ACC. An additional strength is that the alteration of *ZWINT*, *PRC1*, *CDKN3*, *CDK1* and *CCNA2* is significantly associated with worse OS and DFS, indicating that these genes may play important roles in the aggressive malignant phenotypes of ACC. At the same time, several limitations of this study are as follows. First, the data utilized in the study consisted of unbalanced ACC and normal control samples, which were restricted in quantity and downloaded from the GEO database, not generated by new DNA microarrays. Second, the microarray data contained relatively few ACC samples in the public database, and only 76 patients were enrolled from the TCGA cohort with corresponding transcriptome data. Third, prospective cohort was not used in this study. In addition, only the mRNA levels of hub genes are shown in this study, thus further functional works and validated cohorts are needed to verify these findings.

## Conclusion

In conclusion, the present study identifies DEGs and hub genes that may be involved in poor prognosis and recurrence of ACC *in silico*. The transcriptional profiles of *ZWINT*, *PRC1*, *CDKN3*, *CDK1* and *CCNA2* are of prognostic value, and may assist in better understanding the underlying carcinogenesis or progression of ACC. Further studies are required to elucidate the molecular pathogenesis and alterations in signaling pathways of these genes in ACC.

## Ethics Statememt

The ethics approval and consent to participate of the current study was approved and consented by the ethics committee of Fudan University Shanghai Cancer Center.

## Author Contributions

The work presented here was carried out in collaboration among all authors. D-WY, H-LZ, and Y-YQ defined the research theme, discussed analyses, interpretation and presentation. W-HX and JlW drafted the manuscript, analyzed the data, developed the algorithm and interpreted the results. JW co-worked on associated data collection, cohort validation and helped to draft the manuscript. F-NW, H-KW, and D-LC helped to perform the statistical analysis and reference collection. All authors read and approved the final manuscript.

## Funding

This work is supported by Grants from the National Natural Science Foundation of China (No. 81202004, 81802525), Natural Science Foundation of Shanghai (No. 16ZR1406400), and Shanghai Sailing Program of China (No. 17YF1402700).

## Conflict of Interest Statement

The authors declare that the research was conducted in the absence of any commercial or financial relationships that could be construed as a potential conflict of interest.

## Abbreviations

ACC, adrenocortical carcinoma; DEGs, differentially expressed genes; GO, Gene Ontology; KEGG, Kyoto Encyclopedia of Genes and Genomes; PPI, protein-protein interaction; TCGA, the Cancer Genome Atlas; HPF, high power field; DFS, disease-free survival; OS, overall survival; HR, hazard ratio; CI, confidence interval; ROC, receiver operating characteristic curve; AUC, area under curve; GSEA, Gene set enrichment analysis.

## Supplementary Material

Supplementary Figure 1 Functional and pathway enrichment analyses were performed using DAVID in bubble chart. **(A)** Changes in cellular components of DEGs were mainly enriched in the chromosome, centromeric region, extracellular region, actin cytoskeleton and mitotic spindle. **(B)** Changes in molecular funtions were mostly enriched in growth factor binding, kinase activity, extracellular matrix structure constituent and insulin-like grouth factor binding. **(C)** GO analysis results showed that changes in biological processes of DEGs were significantly enriched in mitotic cell cycle, cell cycle process, movement of cell or subcellular component and cell locomotion activity.

Supplementary Figure 2 A network of the 24 genes and their co-expression genes was visualized and displayed in detail. **(A)** The biologic process and KEGG enrichment analysis of the hub genes were shown in different color. **(B)** The detailed functional notes and classification pie charts are listed as follows. 66.67% terms belong to mitotic cell cycle checkpoint, 15.79% to mitotic spindle organization, 12.28% to anaphase-promoting complex-dependent catabolic process, 3.51% to protein localization to kinetochore and 1.75% to chromosome consederation.

Supplementary Figure 3 Univariate survival analysis of the hub genes was performed using Kaplan-Meier curve. Besides MND1, each elevated expression in 24 hub gene showed markedly significant worse OS and DFS in ACC samples (*P*< 0.05).

Supplementary Figure 4 Sensitivity analyze of GSE19750 and GSE90713 with Venn diagram, PPI network and the most significant module of DEGs. **(A)** DEGs were selected with a fold change >2 and P-value <0.01 among the mRNA expression profiling chip datasets GSE19750 and GSE90713. The 2 datasets showed an overlap of 315 genes in Venn diagram. **(B)** The PPI network of DEGs was constructed using Cytoscape. **(C)** The most significant module was obtained from PPI network with 31 nodes including ZWINT, PRC1, CDKN3, CDK1, CCNA2. Significant edges are marked in light blue with a K-score >0.800.

Supplementary Figure 5 Univariate survival analysis of hub genes from sensitivity validated datasets was performed using Kaplan-Meier curve. Eight different DEGs are found different from these in three-chipset study, including *UBE2C*, *GINS1*, *SMC2*, *ATAD2*, *PRIM1*, *NUSAP1*, *SPAG5*, *SMC4*. Kaplan-Meier method was used to analysis mRNA expression level of 8 hub genes in TCGA cohort, which also showed significant correlation between elevated expression and progressive progression or poor prognosis (*P*< 0.05).
