# Supplementary figures and images for: Screening and Identification of Potential Prognostic Biomarkers in Adrenocortical Carcinoma
Source: Front Genet. 2019 Sep 11;10:821. doi: 10.3389/fgene.2019.00821 (PMC6749084; doi:10.3389/fgene.2019.00821)

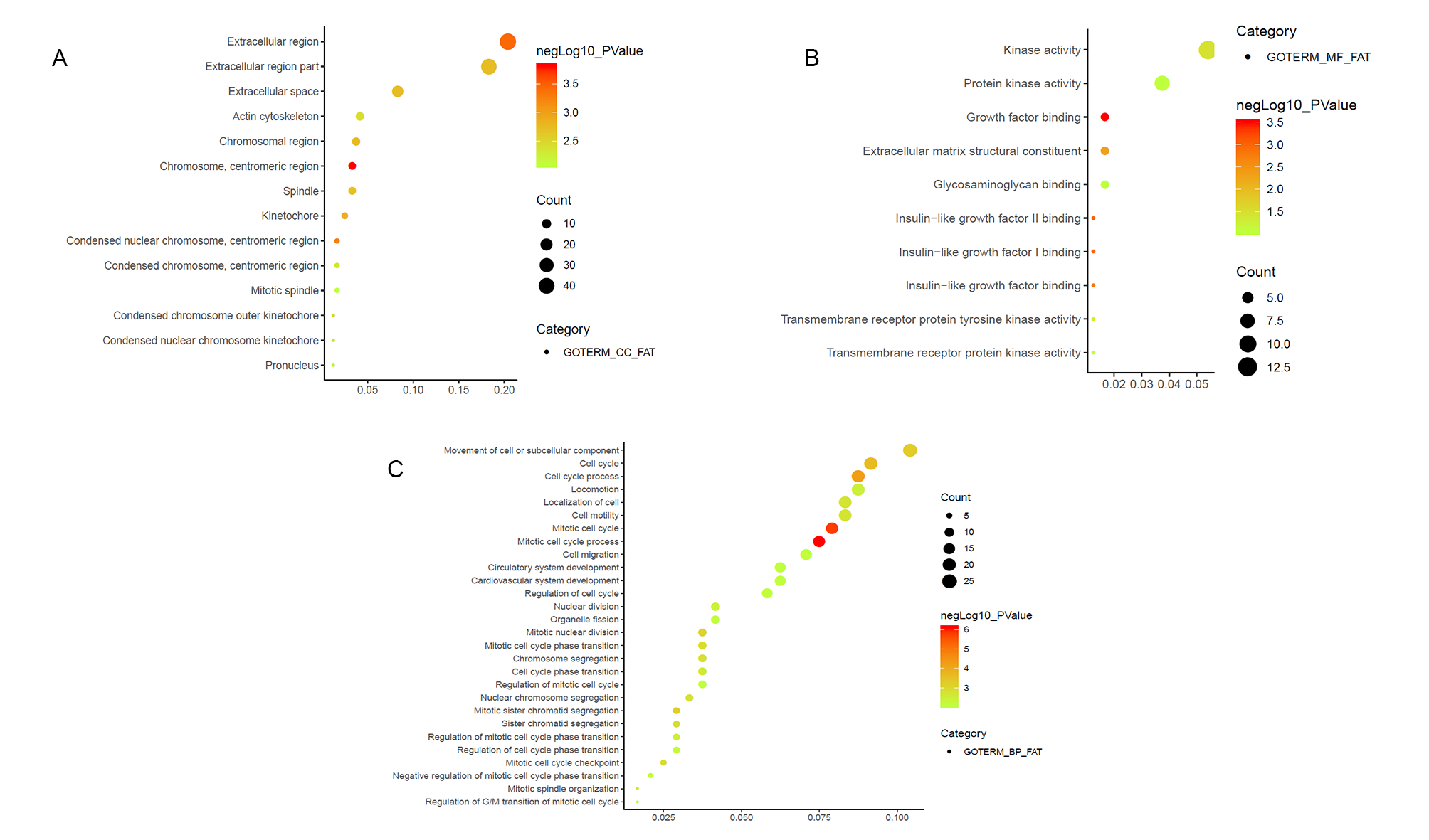

Supplement: Supplementary Figure 1 — Functional and pathway enrichment analyses were performed using DAVID in bubble chart. (A) Changes in cellular components of DEGs were mainly enriched in the chromosome, centromeric region, extracellular region, actin cytoskeleton and mitotic spindle. (B) Changes in molecular funtions were mostly enriched in growth factor binding, kinase activity, extracellular matrix structure constituent and insulin-like grouth factor binding. (C) GO analysis results showed that changes in biological processes of DEGs were significantly enriched in mitotic cell cycle, cell cycle process, movement of cell or subcellular component and cell locomotion activity. [file Image_1.tif]

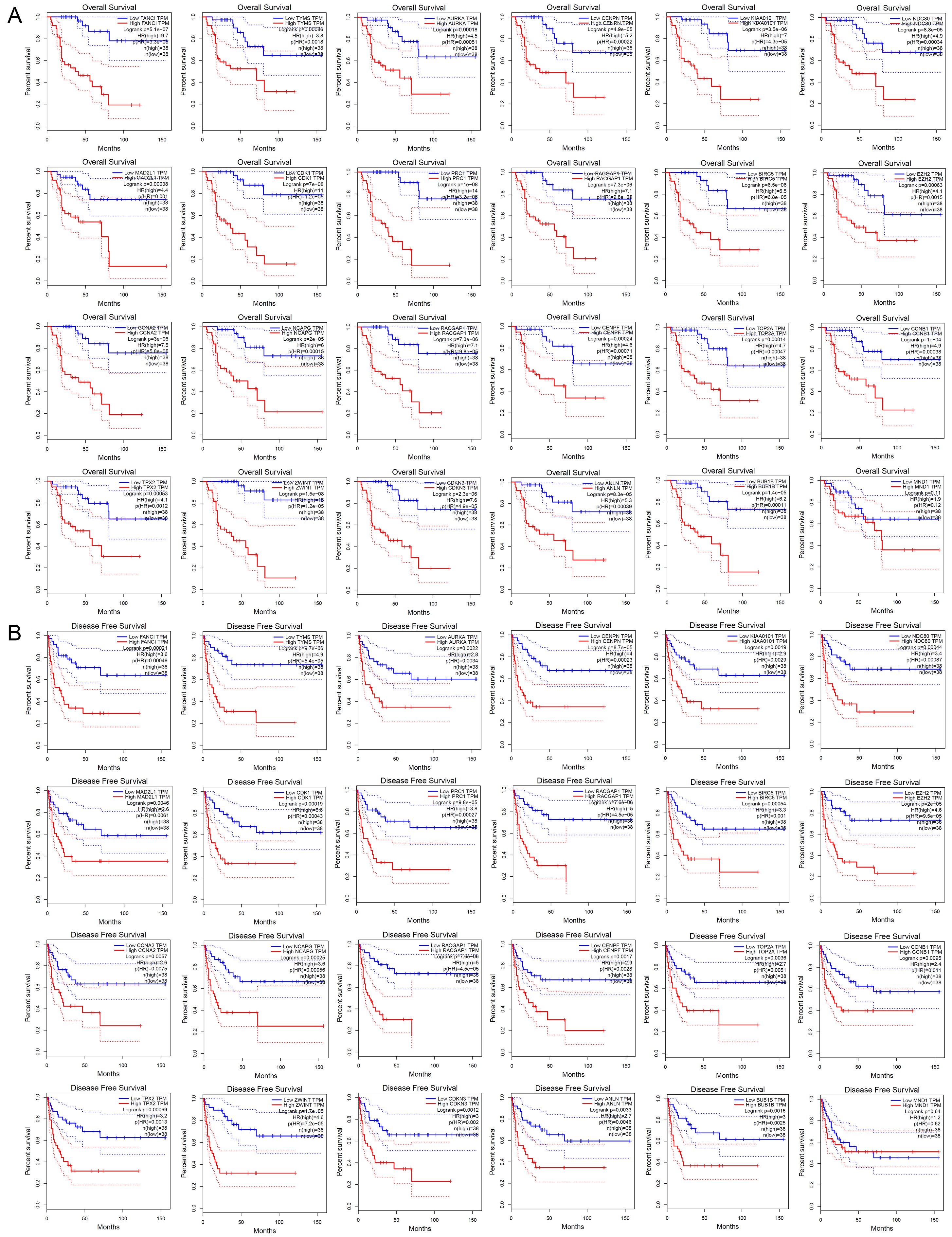

Supplement: Supplementary Figure 2 — A network of the 24 genes and their co-expression genes was visualized and displayed in detail. (A) The biologic process and KEGG enrichment analysis of the hub genes were shown in different color. (B) The detailed functional notes and classification pie charts are listed as follows. 66.67% terms belong to mitotic cell cycle checkpoint, 15.79% to mitotic spindle organization, 12.28% to anaphase-promoting complex-dependent catabolic process, 3.51% to protein localization to kinetochore and 1.75% to chromosome consederation. [file Image_2.tif]

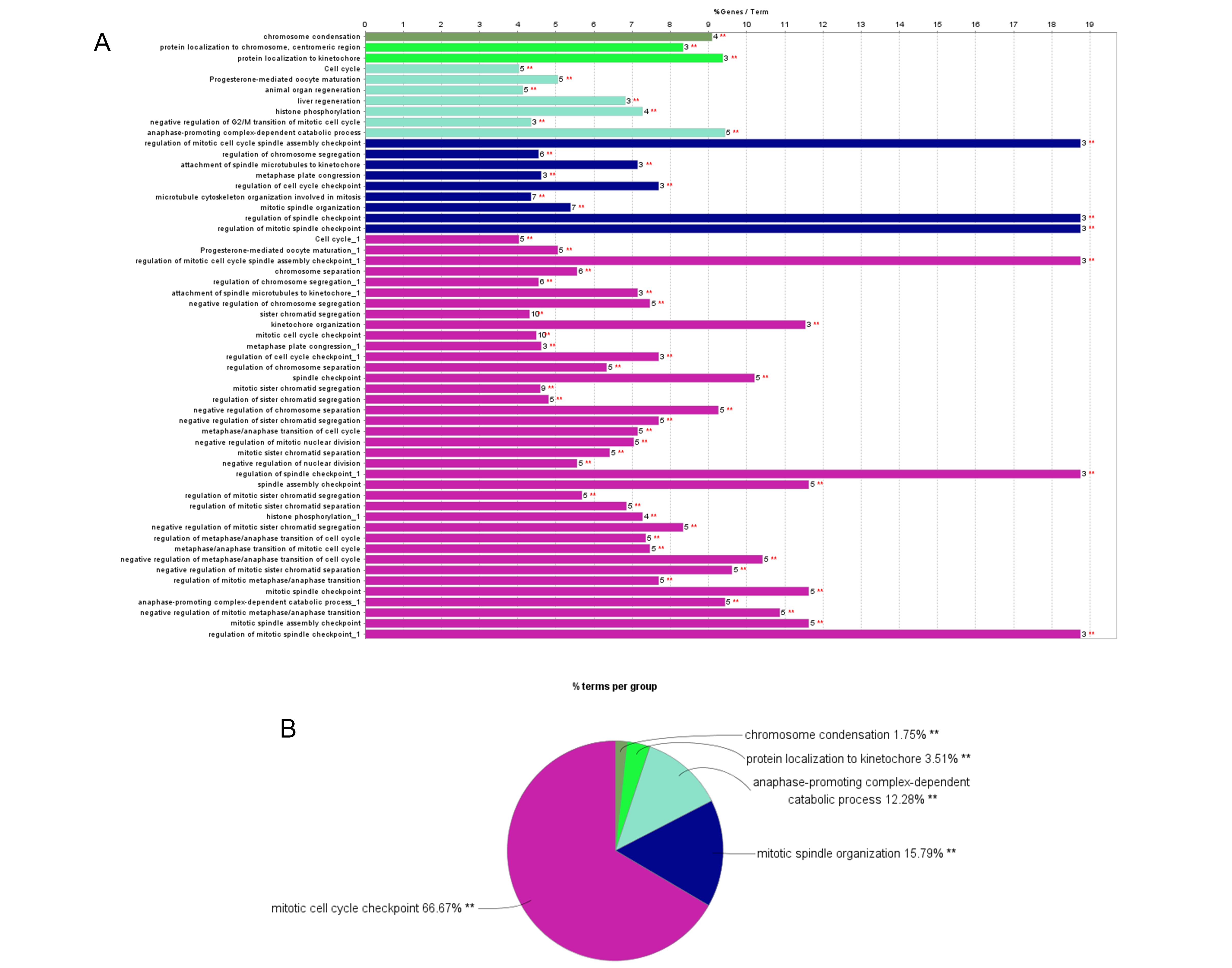

Supplement: Supplementary Figure 3 — Univariate survival analysis of the hub genes was performed using Kaplan-Meier curve. Besides MND1, each elevated expression in 24 hub gene showed markedly significant worse OS and DFS in ACC samples (P< 0.05). [file Image_3.tif]

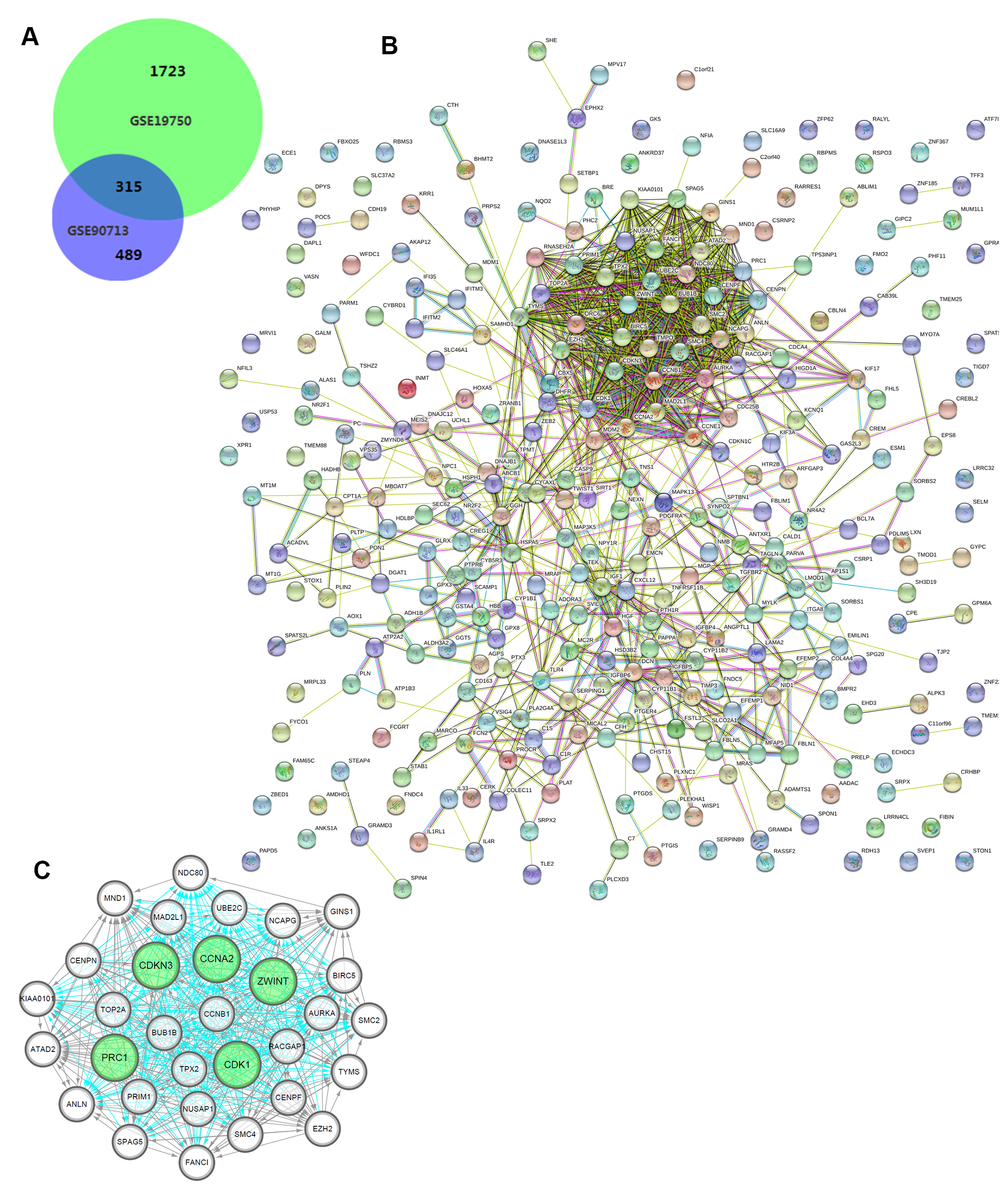

Supplement: Supplementary Figure 4 — Sensitivity analyze of GSE19750 and GSE90713 with Venn diagram, PPI network and the most significant module of DEGs. (A) DEGs were selected with a fold change >2 and P-value <0.01 among the mRNA expression profiling chip datasets GSE19750 and GSE90713. The 2 datasets showed an overlap of 315 genes in Venn diagram. (B) The PPI network of DEGs was constructed using Cytoscape. (C) The most significant module was obtained from PPI network with 31 nodes including ZWINT, PRC1, CDKN3, CDK1, CCNA2. Significant edges are marked in light blue with a K-score >0.800. [file Image_4.tif]

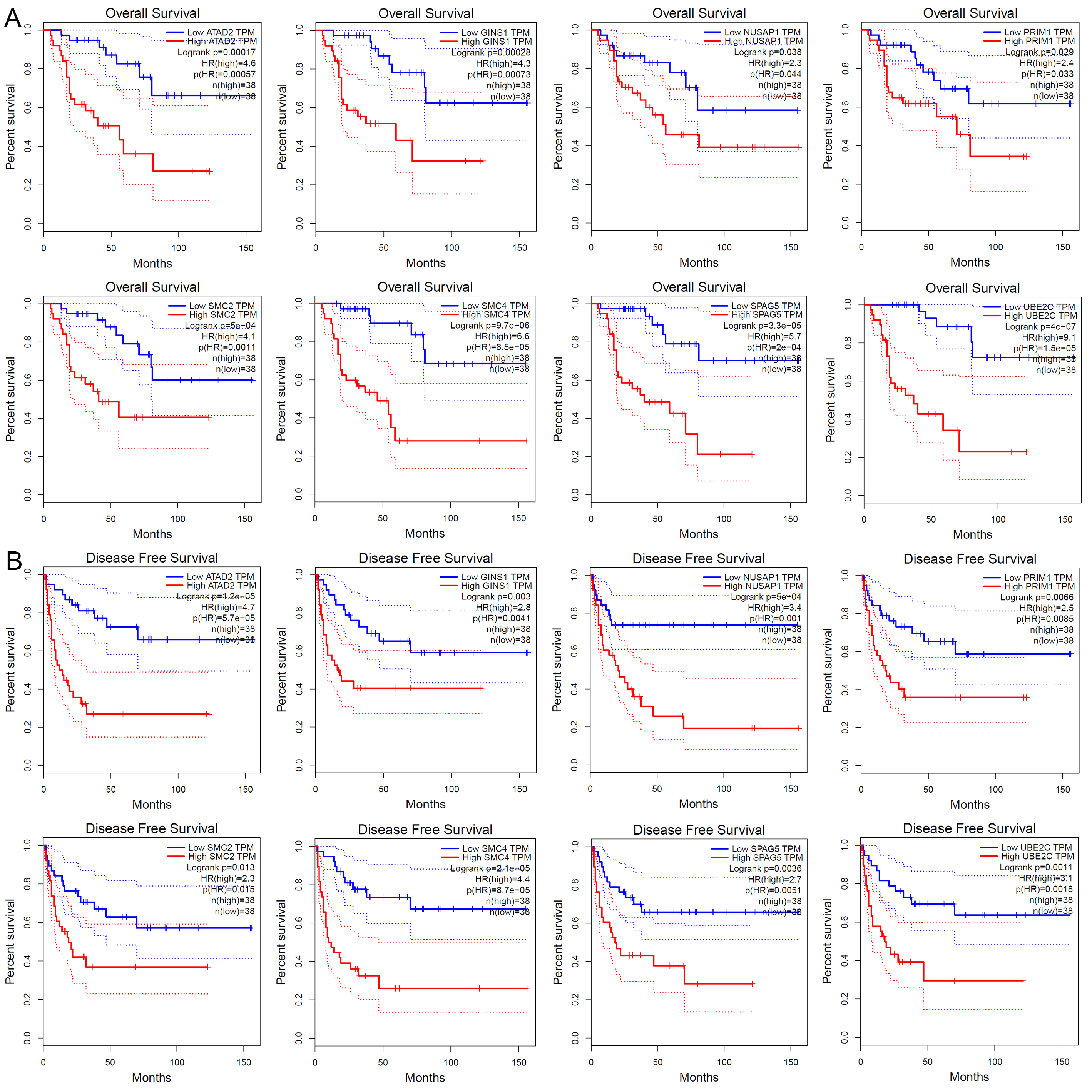

Supplement: Supplementary Figure 5 — Univariate survival analysis of hub genes from sensitivity validated datasets was performed using Kaplan-Meier curve. Eight different DEGs are found different from these in three-chipset study, including UBE2C, GINS1, SMC2, ATAD2, PRIM1, NUSAP1, SPAG5, SMC4. Kaplan-Meier method was used to analysis mRNA expression level of 8 hub genes in TCGA cohort, which also showed significant correlation between elevated expression and progressive progression or poor prognosis (P< 0.05). [file Image_5.tif]
